# Multimodal Imaging of the White Dot Syndromes and Related Diseases

**DOI:** 10.4172/2155-9570.1000570

**Published:** 2016-06-27

**Authors:** Jared E Knickelbein, H Nida Sen

**Affiliations:** National Eye Institute, National Institutes of Health, Bethesda, USA

**Keywords:** Uveitis, White dot syndromes, Multimodal imaging

## Abstract

The white dot syndromes encompass a group of rare posterior uveitis conditions that are characterized by outer retinal and/or choroidal hypopigmented lesions that are thought to be inflammatory in nature. The size, shape, and location of lesions in the fundus aid in differentiating these conditions. Multimodal imaging, including modalities such as fundus autofluorescence, optical coherence tomography, fluorescein angiography, and indocyanine green angiography, among others, has become integral in diagnosing and monitoring many of the white dot syndromes. Furthermore, multimodal imaging modalities have provided insights into the pathogenesis and exact sites within the retina and choroid affected by white dot syndromes.

## Introduction

The white dot syndromes (WDS) comprise a group of diseases characterized by lesions of the outer retina, retinal pigment epithelium, choriocapillaris, choroid or a combination of these sites [1,2]. The WDS are hypothesized to have an inflammatory etiology, and this is more evident for certain conditions, such as birdshot chorioretinopathy (BCR), in which vitreous inflammation and retinal vasculitis often accompany the choroidal birdshot lesions. In many of the other WDS, inflammation of the anterior chamber and vitreous cavity may be lacking. While the WDS often present with symptoms of blurred vision, floaters, photopsias, and scotomata, fundus examination and imaging reveal distinct findings, including the specific location, size, and configuration of the lesions, allowing distinct classification of the disease.

Continuing advancements in ocular imaging technologies now allow for detailed assessment of both the anatomy and function of many posterior segment structures through multimodal imaging. These imaging modalities include fundus autofluorescence (FAF; structure and function of RPE), optical coherence tomography (OCT; cross-sectional view of vitreoretinal and choroidal structure), fluorescein angiography (FA; assessment of retinal vasculature), indocyanine green angiography (ICGA; assessment of choroidal vasculature), as well as wide-field technologies that encompass many of these modalities. Combining the information provided by these various modalities allows the clinician to identify the particular site of pathology aiding in diagnosis and gauge the severity of disease.

Multimodal imaging is often useful in the diagnosis and management of posterior segment disease. This is especially true for WDS. This review will focus on the characteristic multimodal imaging findings for several WDS in which multimodal imaging is especially useful.

## Birdshot chorioretinopathy

**Figure 1:**
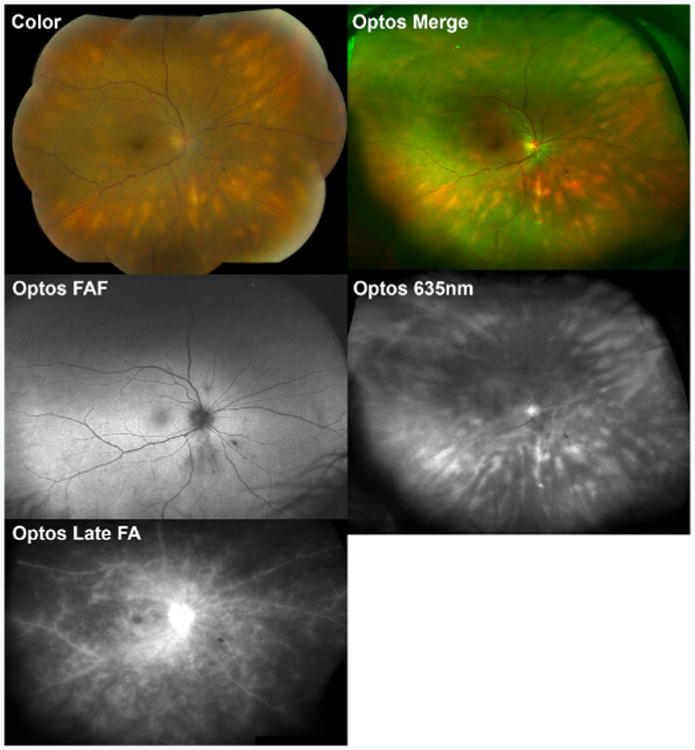
Multimodal imaging of a 63 year-old woman with birdshot chorioretinopathy (BCR). Note the deep hypopigmented lesions in the mid-periphery on color fundus photography (Color) and wide-field Optos pseudocolored (Optos Merge) images. These lesions are especially apparent on Optos 635 nm laser images that focuses on choroidal structures (Optos 635nm). Wide-field Optos fundus autofluorescence (Optos FAF) reveals patchy areas of hypoautofluorescence, some of which do not overlap with underlying birdshot lesions. Wide-field Optos fluorescein angiography reveals diffuse vascular leakage in the late frames (Optos Late FA).

**Figure 2:**
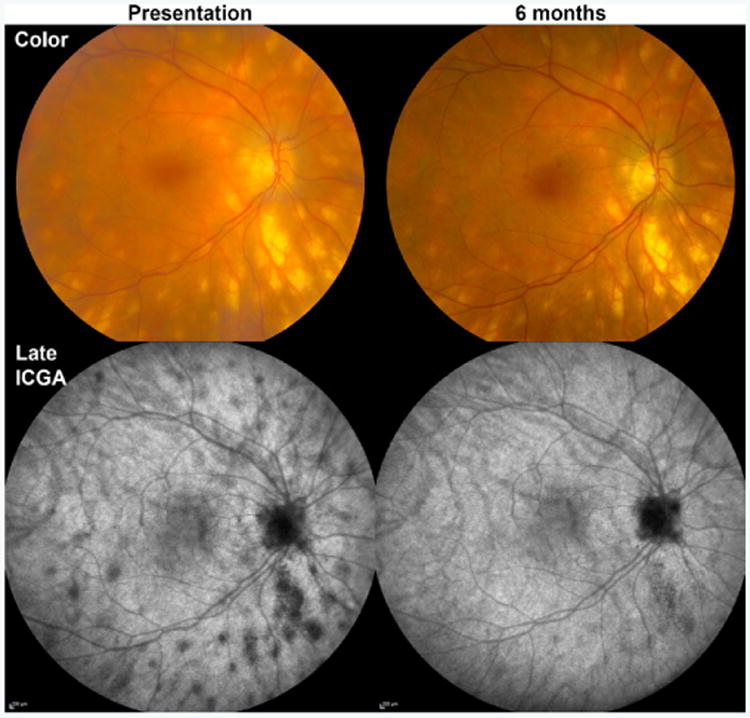
Multimodal imaging of a 53 year-old woman with birdshot chorioretinopathy (BCR). On presentation, the hypopigmented birdshot lesions seen on color fundus photography (Color) appear as hypofluorescent spots on late indocyanine green angiography (Late ICGA). 6 months later, after receiving treatment with oral prednisone and mycophenolate mofetil, the lesions are essentially unchanged on color photographs, while late ICGA reveals substantially fewer dark spots.

Birdshot chorioretinopathy (BCR), which has also been referred to as vitiliginous chorioretinitis, is an inflammatory posterior uveitic syndrome characterized by deep small-to-medium-sized round or oval hypopigmented choroidal lesions radiating from the optic nerve in a pattern similar to birdshot from a shotgun (Figure 1) [3,4]. Birdshot lesions are easily detected by the 635 nm laser on Optos^®^ imaging as perivascular choroidal lesions (Figure 1) [5]. ICGA shows characteristic hypofluorescent choroidal spots that correspond to and are typically greater in number than the clinically apparent lesions during active disease (Figure 2) [6]. Hypoautofluorescent ICGA lesions may resolve with immunosuppressive treatment.

Vitreous inflammation and retinal vasculitis are often present as well, and FA is used to diagnose and monitor the vasculitic component. Furthermore, FA may demonstrate optic nerve hyperfluorescence, macular edema, retinal non-perfusion, or retinal neovascularization in advanced cases. OCT is most useful in evaluating for macular edema, which is the most common cause of vision loss in patients with BCR [7]. FAF abnormalities are not uncommon in birdshot chorioretinopathy, but interestingly, they do not often overlap with underlying choroidal inflammatory birdshot lesions (Figure 1) [8-10]. This suggests that inflammatory damage may be occurring separately in the choroid and overlying outer retinal structures. Macular hypoautofluorescence, indicative of atrophy, is associated with poor visual acuity [9].

## Acute posterior multifocal placoid pigmentary epitheliopathy

Acute posterior multifocal placoid pigmentary epitheliopathy (APMPPE) is characterized by large plaque-like posterior pole lesions at the level of RPE and inner choroid [11]. APMPPE generally affects young (typically <30 years but can be older) otherwise healthy individuals often following a viral illness [2]. APMPPE seems to develop in women and men equally and is usually, but not always, bilateral. Lesions may resolve spontaneously without treatment; however, in more severe cases, outer retinal disruption with corresponding scotomata is permanent.

**Figure 3:**
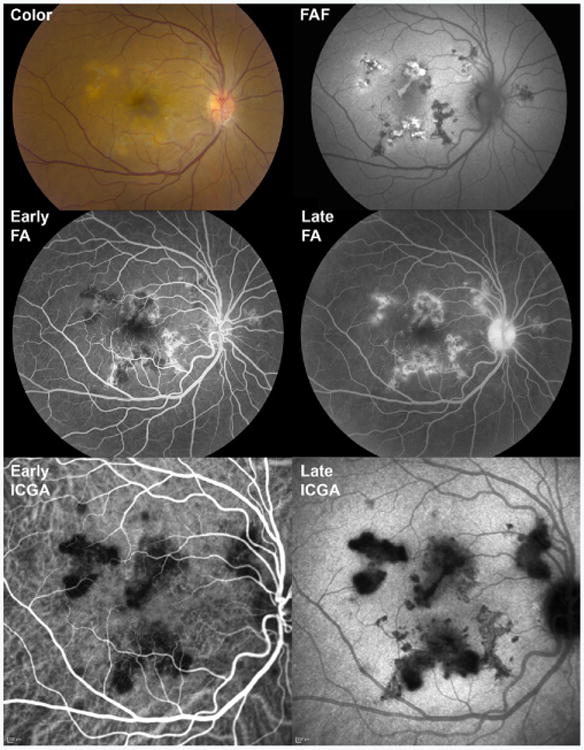
Multimodal imaging of a 51 year-old woman with acute posterior multifocal placoid pigment epitheliopathy (APMPPE). At presentation, color fundus photography (Color) showed large hypopigmented placoid lesions in the macula. On fundus autofluorescence (FAF), some lesions were hypoautofluorescent (likely atrophic), some mixed hyper- and hypo-autofluorescent, and some had a surrounding hyperautofluorescent halo (likely active). On fluorescein angiography (FA), lesions were mostly hypofluorescent during early frames (Early FA) with staining on later frames (Late FA). On indocyanine green angiography, lesions were hypofluorescent early (Early ICGA) with persistence and expansion of hypofluorescence in late frames (Late ICGA).

During the acute phase of the disease, APMPPE presents with creamy deep placoid lesions on fundoscopy (Figure 3). FA during acute disease shows early hypofluorescence with progressive irregular staining of lesions (Figure 3) [11]. ICGA shows early hypofluorescence that persists into the late frames (Figure 3) and often is larger in area than the visible lesions on exam [12]. OCT demonstrates outer retinal and RPE disruption corresponding to the lesions, and in some cases, outer retinal cysts have been described [13,14]. FAF imaging may reveal a spectrum of abnormalities ranging from mostly hypoautofluorescent lesions to a mottled signal of mixed hyper- and hypo-autofluorescence, at times with a hyperfluorescent halo surrounding the lesion (Figure 3) [9,15,16]. Debate continues as to whether the primary site of pathology lies in the RPE or choriocapillaris (i.e. early hypofluorescence on FA may be due to blockage from inflamed RPE or choriocapillaris non-perfusion). As suggested by ICGA studies twenty years ago, [17] emerging studies using OCT angiography (OCTA) have demonstrated choriocapillaris non-perfusion in APMPPE [18].

**Figure 4:**
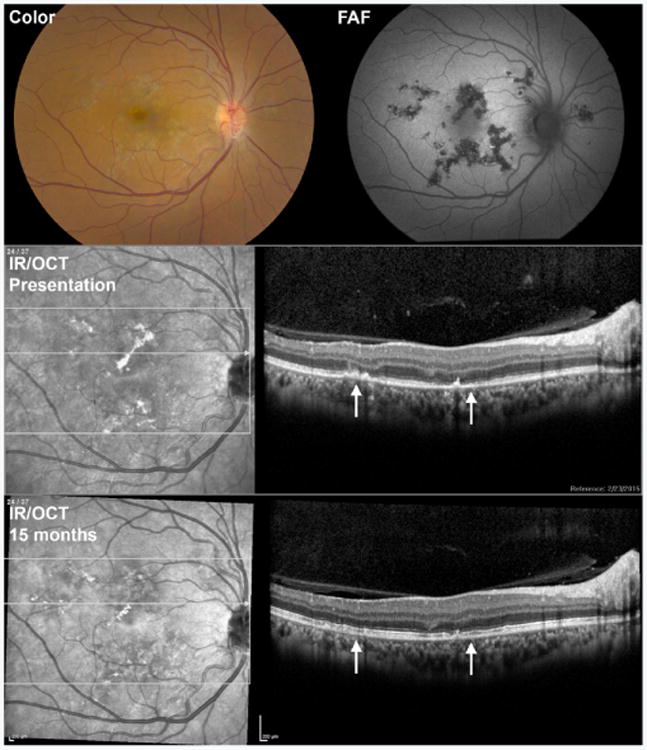
Multimodal imaging of a 51 year-old woman with acute posterior multifocal placoid pigment epitheliopathy (APMPPE). Color fundus photography (Color) and fundus autofluorescence (FAF) images are from the same patient as in figure 3, 15 months following presentation and after receiving a short course of oral prednisone. Note the pigmentary changes in areas of the prior placoid lesions on color images. On FAF 15 months after presentation, lesions were mostly hypoautofluorescent, suggesting atrophy. At presentation, optical coherence tomography (OCT) revealed outer retinal and RPE disruption corresponding to the lesions. Some restructuring of the outer retinal architecture is apparent 15 months after presentation. Infrared reflectance (IR) images are shown to demark the location of OCT B scans.

The acute phase of APMPPE generally resolves over the course of weeks to months [1,2]. Smaller lesions may resolve spontaneously with little evidence of prior pathology on ophthalmoscopy. However, larger lesions often evolve into areas of coarse deep retinal pigmentation (Figure 4). Areas of ICGA hypofluorescence, both early and late, generally improve with time. OCT may show some reconstitution of outer retinal structures, but FAF often remains abnormal in the area of previous lesions (Figure 4). OCT and FAF are especially useful in monitoring APMPPE because they provide rapid non-invasive assessment of lesion size and activity.

## Serpiginous choroiditis

Serpiginous choroiditis, also known as geographic helicoid peripapillary choroidopathy, classically presents with helicoid or geographic white-to-grey peripapillary lesions that extend outward from the disc in a serpentine configuration [19,20]. Vision is lost when the fovea becomes involved. A variant referred to as macular serpiginous choroiditis presents with similar lesions predominantly affecting the macula, often with a poorer prognosis given the high frequency of foveal involvement [21,22]. Serpiginous choroiditis is characterized by periods of disease activity and remission with recurrent activity often occurring at the edge of previous lesions [19,20]. With time, lesions evolve into chorioretinal atrophy. Given the recurrent nature of the disease, lesions often appear to have areas in multiple stages of activity with some areas appearing more active or atrophic than others. Serpiginous choroiditis has been associated with tuberculosis (TB) infection in endemic areas [23]. In patients who are not infected with TB, serpiginous choroiditis is thought to be driven by an autoimmune inflammatory reaction, and immunosuppression is often employed in an attempt to preserve vision by halting progression and preventing recurrent disease [24].

**Figure 5:**
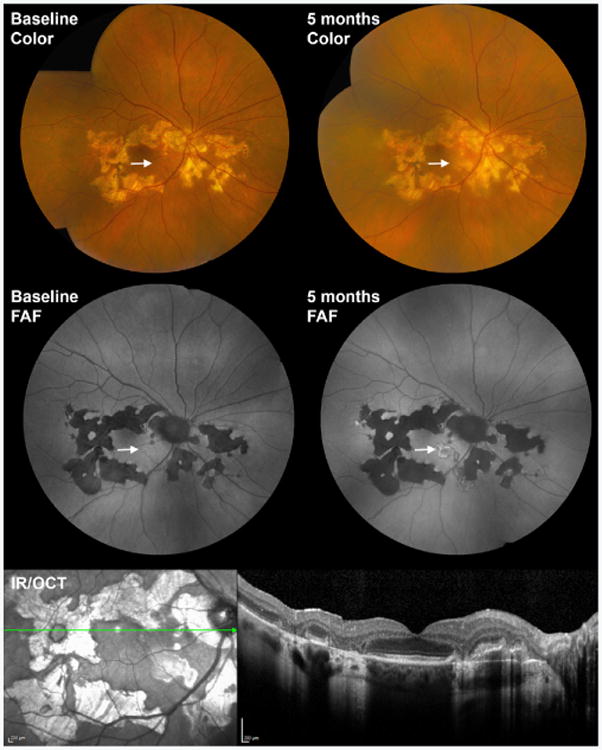
Multimodal imaging of a 67 year-old man with serpiginous choroiditis. During a period of quiescence, color fundus photography demonstrates a helicoid pattern of chorioretinal atrophy emanating from the optic nerve (Baseline Color), while fundus autofluorescence shows corresponding areas of hypoautofluorescence (Baseline FAF). 5 months later, despite immunosuppressive therapy, disease activity returned with a new area of retinal whitening on color fundus photography (5 months Color, arrow). The newly active lesion appeared mostly hyperautofluorescent on FAF. Optical coherence tomography demonstrated outer retinal loss and RPE disruption in the areas of lesions. An infrared reflectance (IR) image is shown to demark the location of OCT B scans.

**Figure 6:**
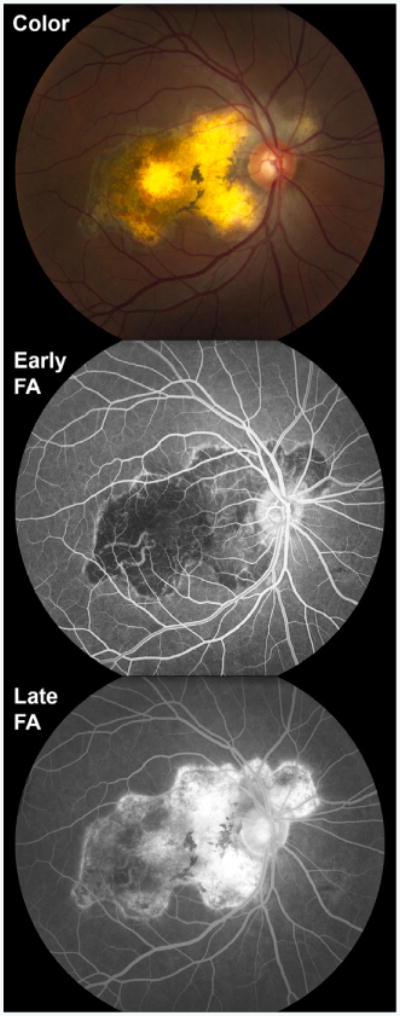
Multimodal imaging of a 44 year-old man with serpiginous choroiditis. Color fundus photography shows a well-demarcated, geographic, white-to-yellow, chorioretinal lesions surrounding the optic nerve. Fluorescein angiography (FA) demonstrates hypofluorescence during the early frames (Early FA) with staining in late frames (Late FA).

During active disease, serpiginous choroiditis typically presents with large peripapillary hypopigmented helicoid lesions extending outward from the disc in a serpentine configuration (Figure 5) [24]. FAF imaging reveals hypoautofluorescence in areas of atrophy, while more active areas are typically hyperautofluorescent (Figure 5) [9,25,26]. OCT shows disruption of the outer retinal structures and RPE (Figure 5). Intra- and sub-retinal fluid may be present, especially at the edges of active lesions. FA shows early hypofluorescence with progressive staining of the lesions later in the angiogram (Figure 6). As with APMPPE, OCT and FAF are especially useful in monitoring serpiginous choroiditis because they provide rapid non-invasive assessment of lesion size and activity [9].

## Ampiginous chorioretinitis

Ampiginous chorioretinitis, also known as relentless placoid chorioretinitis, presents with extensive deep amoeboid-shaped lesions similar to APMPPE but has a progressive course akin to serpiginous chorioretinitis with lesions often extending through the posterior pole and into the peripheral retina [2,27]. As in APMPPE, a viral prodrome is often observed; however, the precise pathogenesis of this disease is unknown. Given the severe presentation of these patients, immunosuppression with systemic corticosteroids is often employed during acute disease and long-term steroid-sparing therapy may be required [2].

**Figure 7:**
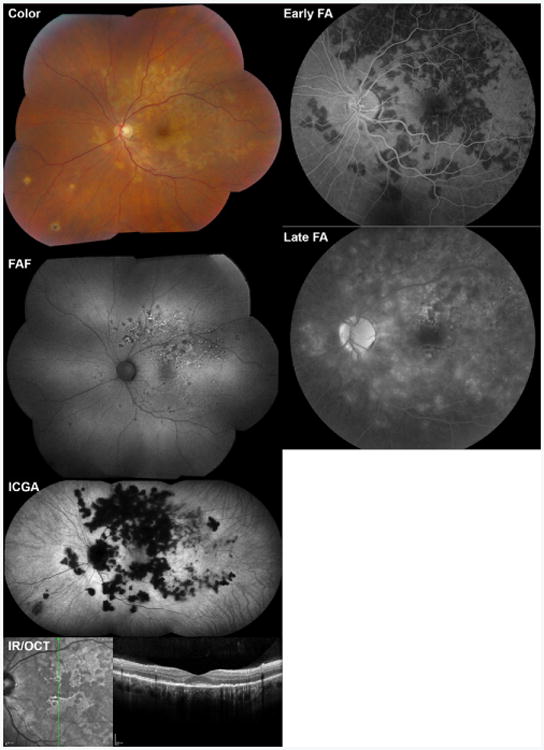
Multimodal imaging of a 49 year-old woman with ampiginous chorioretinitis. At presentation, color fundus photography (Color) showed an extensive network of hypopigmented lesions in the macula and mid-periphery. On fundus autofluorescence (FAF), some lesions were hypoautofluorescent (likely atrophic), some mixed hyper- and hypo-autofluorescent, and some had a surrounding hyperautofluorescent halo (likely active). On fluorescein angiography (FA), lesions were mostly hypofluorescent during early frames (Early FA) with staining on later frames (Late FA). On indocyanine green angiography, lesions were hypofluorescent early (not shown) with persistence and expansion of hypofluorescence in late frames (ICGA). Optical coherence tomography demonstrates outer retinal loss and RPE disruption in the areas of lesions. Infrared reflectance (IR) images are shown to demark the location of OCT B scans.

During acute disease, ampiginous lesions appear as creamy deep amoeboid-shaped lesions, similar to those in APMPPE, but often dispersed throughout the posterior pole and extending into the retinal periphery (Figure 7). OCT shows outer retinal and RPE disruption corresponding to the lesions (Figure 7). Intra- and sub-retinal fluid may be present, especially at the edges of active lesions. FAF demonstrates a network of abnormal signal with areas of mixed hypofluorescence, often at the center of lesions, and hyperfluorescence, often at the lesion borders (Figure 7). FA shows early hypofluorescence with late staining. During convalescence, lesions often contract and become more pigmented on fundoscopy (Figure 7). On FAF, convalescent lesions appear more hypoautofluorescent reflecting permanent RPE derangement. As with APMPPE and serpiginous chorioretinitis discussed above, OCT and FAF imaging are useful for both diagnosis and monitoring of ampiginous chorioretinitis.

## Multifocal choroiditis and panuveitis & punctate inner choroidopathy

Multifocal choroiditis and panuveitis (MCP) is an idiopathic panuveitis characterized by discrete, variably sized, chorioretinal lesions throughout the fundus accompanied by an inflammatory reaction in the vitreous cavity and often in the anterior chamber. Punctate inner choroidopathy (PIC) is an uncommon idiopathic inflammatory chorioretinopathy characterized by multiple, small (100-300 μm), discrete, yellow-white, posterior pole lesions [2,28]. Patients with PIC generally lack signs of inflammation in the anterior chamber and vitreous. MCP and PIC are more common in young moderately myopic females and are generally bilateral. Choroidal neovascularization (CNV) may develop and is an important cause of vision loss in MCP and PIC. Given these similarities, MCP and PIC may represent a spectrum of disease rather than independent disease processes [29]. Furthermore, disruption of outer retinal structures on OCT, similar to that seen in acute zonal occult outer retinopathy (described below), beyond the individual chorioretinal lesions has been reported in cases of MCP and PIC [30-32].

**Figure 8:**
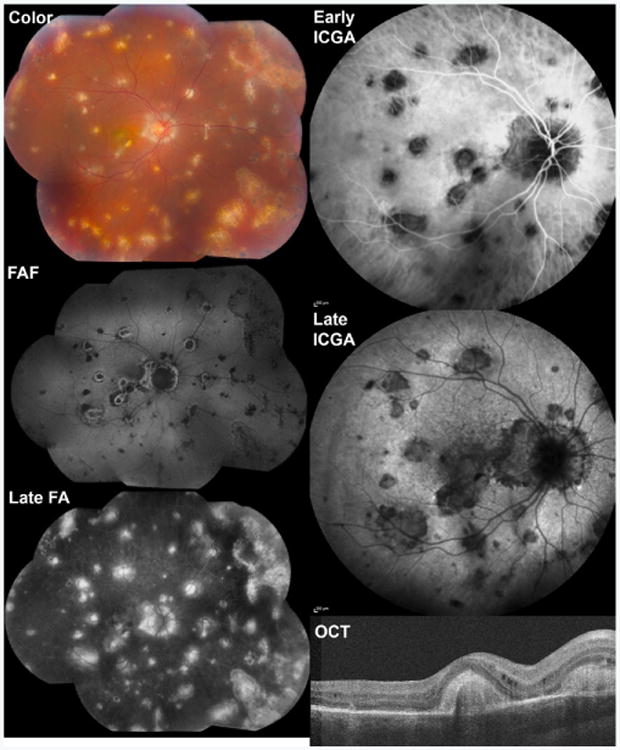
Multimodal imaging of a 29 year-old woman with multifocal choroiditis and panuveitis (MCP). Color fundus photography (Color) demonstrates round chorioretinal lesions of various size scattered throughout the macula and periphery. On fundus autofluorescence (FAF), some lesions were hypoautofluorescent (likely atrophic), some mixed hyper- and hypo-autofluorescent, and some had a surrounding hyperautofluorescent halo (likely active). Fluorescein angiography (FA) showed early hypofluorescence (not shown) with late staining (Late FA). On indocyanine green angiography (ICGA), lesions were hypofluorescent early (Early ICGA) with persistence and expansion of hypofluorescence in late frames (Late ICGA). Optical coherence tomography (OCT) showed hyper-reflective outer retinal nodular lesions corresponding to clinically apparent lesions with more widespread disruption in the surrounding outer retinal architecture.

The chorioretinal lesions in MCP and PIC may have indistinguishable features on multimodal imaging [29]. FAF imaging of active lesions tends to show a hypoautofluorescent center with surrounding hyperautofluorescence (Figure 8) [9,33,34]. Lesions become more uniformly hypoautofluorescent once scared and inactive. FAF may show lesions not yet apparent by ophthalmoscopy [35]. Therefore, FAF is an important imaging tool in monitoring disease progression in patients with MCP. OCT typically shows hyper-reflective outer retinal nodular lesions corresponding to clinically apparent lesions with more widespread disruption in the surrounding outer retinal architecture (Figure 8) [29]. FA often shows early hypofluorescence with late staining (Figure 8). On ICGA, lesions are typically hypofluorescent early with persistence and expansion of hypofluorescence in late frames (Figure 8). OCT and FA are also useful in cases when CNV is suspected.

## Multiple evanescent white dot syndrome

**Figure 9:**
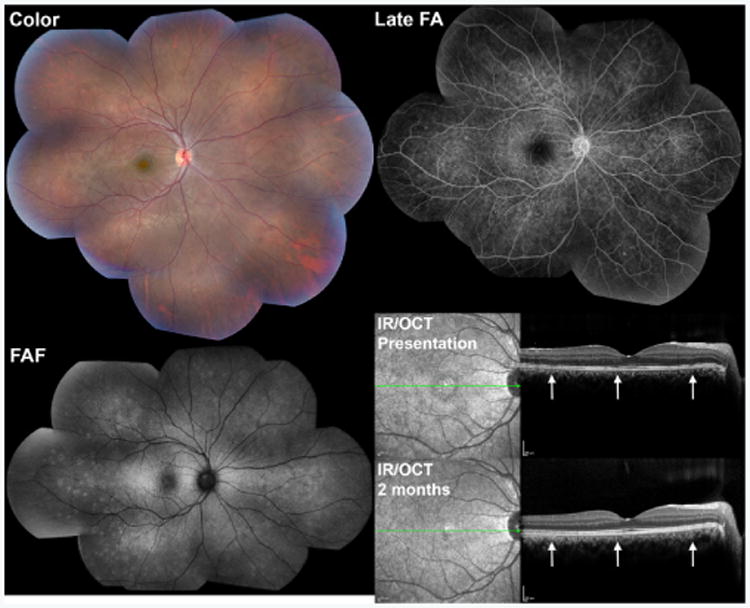
Multimodal imaging of a 17 year-old young man with multiple evanescent white dot syndrome (MEWDS). Note the multiple outer retinal white dots and foveal granularity on the color fundus photograph (Color). On fundus autofluorescence (FAF), lesions were hyperautofluorescent. Fluorescein angiography (FA) showed characteristic “wreath-like” hyperfluorescence of individual lesions that persisted into late frames (Late FA). Optical coherence tomography (OCT) showed disruption of the ellipsoid zone (arrows) corresponding to individual lesions at presentation (OCT Presentation) with reconstitution of these areas two months later after a course of oral prednisone (OCT 2 months). Infrared reflectance (IR) images are shown to demark the location of OCT B scans.

Multiple evanescent white dot syndrome (MEWDS) is an idiopathic, presumed inflammatory condition characterized by multiple outer retinal white dots and foveal granularity (Figure 9) [36]. MEWDS is typically unilateral, affects women more commonly than men, and is often preceded by a viral prodrome. This disorder is commonly transient with resolution of visual symptoms and retinal anatomy over the course of weeks to months.

MEWDS lesions are typically hyperautofluorescent on FAF imaging (Figure 9) [37,38]. FA shows characteristic “wreath-like” hyperfluorescence of lesions that persists into late frames (Figure 9). ICGA typically shows hypofluorescent spots in late frames [39]. OCT shows disruption of photoreceptors, specifically the ellipsoid zone and outer segments, in areas corresponding to individual lesions (Figure 9) [25,38,40]. Reconstitution of outer retinal architecture may be seen with convalescence.

## Acute zonal occult outer retinopathy

Acute zonal occult outer retinopathy is a rare condition characterized by acute zonal loss of outer retinal function with minimal ophthalmoscopic findings [41]. Photopsias and scotomata are commonly experienced by patients with AZOOR, and this condition predominantly affects young women. Electroretinography is often abnormal. The pathogenesis has been suggested to involve viral infection of the RPE with subsequent inflammatory insult.

**Figure 10:**
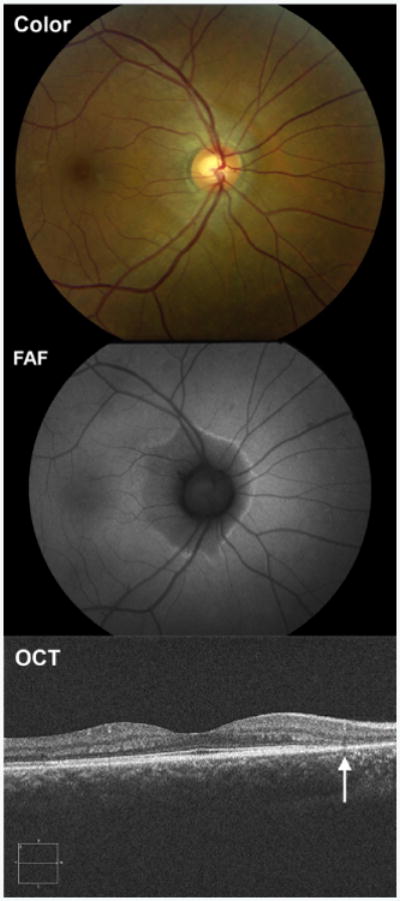
Multimodal imaging of a 35 year-old woman with acute zonal occult outer retinopathy (AZOOR). Color fundus photography (Color) revealed subtle peripapillary pigmentary abnormalities. Fundus autofluorescence (FAF) showed a sharply demarcated area of hypoautofluorescence with a hyperautofluorescent border. Optical coherence tomography (OCT) showed disruption of the outer retinal structures, including the ellipsoid zone and photoreceptor outer segments (arrow), in the area of FAF abnormalities.

Fundoscopy is often normal or may reveal subtle pigmentary abnormalities, often in the peri-papillary region (Figure 10). FAF findings can be variable ranging from normal, to areas of stippled hyper- and hypo-autofluorescence, to areas of sharply demarcated hypoautofluorescence (Figure 10) [42]. FA typically shows early hyperfluorescence from transmission defect that fades in later frames [43]. OCT of affected areas shows disruption of outer retinal structures, including the ellipsoid zone and photoreceptor outer segments (Figure 10).

## Conclusions

As described above, many WDS have characteristic findings on multimodal imaging that aid in distinguishing the specific WDS. Additionally, multimodal imaging is important in monitoring for progression and response to treatment in several of these conditions. Further advances in imaging technology, such as OCT angiography and adaptive optics in combination with either confocal scanning laser ophthalmoscopy or OCT, will undoubtedly provide additional information on the anatomic and functional nature of WDS.
